# A case report of the coagulation management of an adult with moderate haemophilia A undergoing transcatheter aortic valve implantation

**DOI:** 10.1093/ehjcr/ytag484

**Published:** 2026-07-10

**Authors:** Vincent Roger Gianolli, Erica Terazzi, Chiara Trotti, Eugenia Biguzzi, Angelo Di Simone, Andrea Menafoglio, Andrea Demarchi

**Affiliations:** Department of Internal Medicine, Ospedale San Giovanni, via Gallino 12, 6500 Bellinzona, Switzerland; Department of Internal Medicine, Ospedale San Giovanni, via Gallino 12, 6500 Bellinzona, Switzerland; Department of Haematology, Istituto Oncologico Della Svizzera Italiana (IOSI), Ospedale San Giovanni, via Gallino 12, 6500 Bellinzona, Switzerland; Department of Haematology, Istituto Oncologico Della Svizzera Italiana (IOSI), Ospedale San Giovanni, via Gallino 12, 6500 Bellinzona, Switzerland; Cardiocentro Ticino Institute, Ente Ospedaliero Cantonale, via Tesserete 48, 6900 Lugano, Switzerland; Cardiocentro Ticino Institute, Ente Ospedaliero Cantonale, via Tesserete 48, 6900 Lugano, Switzerland; Cardiocentro Ticino Institute, Ente Ospedaliero Cantonale, via Tesserete 48, 6900 Lugano, Switzerland

**Keywords:** Aortic stenosis, Haemophilia A, TAVI, Case report

## Abstract

**Background:**

Haemophilia A is an X-linked bleeding disorder resulting from factor VIII deficiency. While surgical management requires the correction of factor VIII levels by the administration of factor VIII concentrate, antiplatelet therapy after lower-risk cardiac surgery/procedures (such as transcatheter aortic valve implantation—TAVI) is not yet standardized. This report describes the management of a patient with moderate haemophilia A undergoing TAVI for severe aortic stenosis.

**Case summary:**

A 76-year-old man with untreated haemophilia A was admitted with traumatic bleeding. After initial stabilization, he developed acute heart failure due to newly discovered severe aortic stenosis. TAVI procedure was performed by transfemoral access according to multidisciplinary team-discussion; a bio-prosthetic valve was successfully implanted. We outline the peri-procedural haemostatic management, which included factor VIII replacement therapy to achieve guideline-recommended activity levels, followed by progressive dose reduction. In view of the high bleeding risk and absence of significant coronary artery disease, antiplatelet therapy was limited to a short course. The procedure was successful, with no bleeding complications.

**Discussion:**

In our patient, replacement therapy with rFVIII was initiated with a target FVIII activity between 80 and 100 IU/dL. During the procedure, unfractionated heparin was administered to achieve an ACT target 29 of 200 s. Post-operatively, FVIII replacement therapy was maintained above 60 IU/dL for the first 30-72 h, followed by 40-50 IU/dL from day 4 to day 7. Subsequently, it was decided to continue low-dose FVIII replacement for 32 additional 7 days. This strategy led to a successful management of both cardiological and hematological aspects.

**Conclusion:**

Although evidence is limited to case reports, TAVI appears to be feasible and safe in patients with haemophilia A. Maintaining high factor VIII activity levels and minimizing antithrombotic exposure through multidisciplinary evaluation can effectively reduce haemorrhagic risk. Our case, in line with previous reports, resulted in no bleeding complications.

Learning points

- TAVI is a suitable option in patients with high risk of bleeding due to haemophilia.
- Patients with haemophilia A can safely undergo TAVI with appropriate peri-procedural correction of factor VIII levels.
- Individualized antithrombotic strategies and close multidisciplinary collaboration are essential to reduce bleeding risk while maintaining procedural safety.

## Introduction

Haemophilia A (HA) is an X-linked recessive inherited bleeding disorder caused by a congenital deficiency of factor VIII (FVIII), resulting in prolonged and excessive bleeding either spontaneously or from trauma. There are three grades of severity for this pathology, reflecting the FVIII clotting activity (FVIII:C): severe (FVIII:C < 1 IU/dl), moderate (FVIII:C 1–4 IU/dl), and mild (FVIII:C 5–40 IU/dl). A recent evaluation of registry data from Australia, Canada, France, Italy, New Zealand and the United Kingdom found a prevalence of 24.6 cases per 100 000 newborn males (with approximately one third of patients affected by the severe form).^1–4^

According to the 2021 Task Force for the management of valvular heart disease of the European Society of Cardiology (ESC) and the European Association for Cardio-Thoracic Surgery (EACTS), transcatheter aortic valve implantation (TAVI) is a class I-recommended intervention in symptomatic patients with severe high-gradient aortic stenosis who are older (>75 years old), or in high-risk patients (STS-PROM/EuroSCORE II >8%), or those unsuitable for surgery. After TAVI, lifelong single antiplatelet therapy (SAPT) is recommended in patients with no baseline indication for oral anticoagulation.^5^

TAVI entails multiple invasive steps, each conferring a bleeding risk. Pre-procedural coronary angiography requires arterial access and catheterization, representing an initial source of bleeding risk.^6^ Temporary transvenous pacemaker insertion for pacing during TAVI is routine, but introduces bleeding risks ranging from local haematoma to rare cardiac perforation and acute tamponade. Approximately 5.2% of TAVI patients experience major bleeding within 30 days, most often due to cardiac tamponade (38.4%) or femoral access injury (32.3%).^7^ According to Baldi *et al.*, 11.9% of TAVI recipients required permanent pacemaker implantation for post-procedural conduction disturbances, with 42.4% of cases attributed to persistent third-degree atrioventricular block.^8^ The implantation procedure itself represents an additional invasive step that may increase haemorrhagic risk. The incidence of developing a pocket haematoma, after cardiac electronic device implantation, is around 1%–9.5%.^9^

HA confers a degree of ‘natural anticoagulation’ proportional to the FVIII deficiency. Laboratory studies about thrombin generation in haemophiliac patients show that FVIII/FIX levels <20 IU/dl are associated with a significant reduction of thrombin production, analogous to the effect of therapeutic anticoagulation in non-haemophiliacs.^10,11^ Patients with FVIII <10 IU/dl may be as ‘anticoagulated’ as those on vitamin K antagonists. However, when FVIII levels are higher (>20 IU/dl, as in mild HA or during replacement therapy), their thrombotic risk approaches that of the general population, and standard indications for antithrombotic therapy can be applied. It is well known that patients with HA may develop neutralizing anti–FVIII inhibitors, for which options exist, such as immune tolerance induction, bypassing agents or emicizumab; however, this is beyond the scope of the present case report.

Most evidence regarding antithrombotic therapy in patients with HA is derived from expert opinion and consensus statements rather than randomized trials. Current expert consensus recommends avoiding any antithrombotic therapy in severe HA without prophylaxis, or in patients with inhibitors not on emicizumab. It is recommended to maintain FVIII/FIX levels of 1–5 IU/dl for SAPT, 20 IU/dl for double antiplatelet therapy or oral anticoagulation, and 80 IU/dl for triple therapy. Treatment decisions should be based on the lowest measured FVIII level. Routine proton-pump inhibitor prophylaxis is recommended in all individuals receiving antiplatelet therapy; furthermore, biological valves are generally favoured, as they eliminate the requirement for lifelong anticoagulant therapy.^10,11^

For major surgeries (e.g. cardiovascular procedures), the current guidelines of the World Federation of Haemophilia (WFH) recommend maintaining FVIII activity between 80 and 100 IU/dl pre-operatively. Post-operatively, target FVIII levels should be maintained at 60–80 IU/dl on days 1–3, 40–60 IU/dl on days 4–6, and 30–50 IU/dl on days 7–14.^2^ The management of TAVI in patients with coagulation disorders remains unclear, as there are no specific guidelines. In this article, we present the case of a patient with moderate HA undergoing TAVI for severe symptomatic aortic stenosis.

## Summary figure

**Figure:**
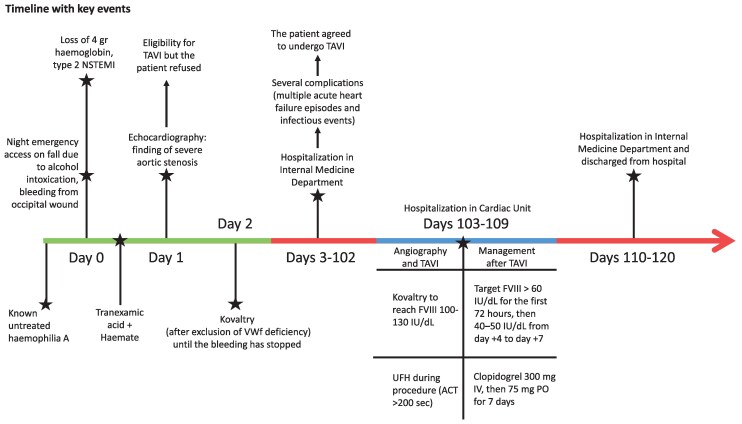
HA, haemophilia A; FVIII, factor VIII; VWF, von Willebrand factor; TAVI, transcatheter aortic valve implantation; Haemate, concentrate with recombinant FVIII plus VWF; Kovaltry, recombinant FVIII concentrate; UFH, unfractioned heparin; ACT, activated clotting time; IV, intravenous; PO, per os.

## Case presentation

A 76-year-old patient was admitted to the emergency department after falling on alcohol intoxication, with extensive bleeding from an occipital wound requiring suturing. He reported HA, not on regular follow-up (last evaluation >40 years before). Any documentation regarding the bleeding disorder was not available, so the severity was unknown, and the patient did not recall any treatment for HA. Physical examination revealed no signs suggestive of haemophilic arthropathy and did not identify overt cardiac abnormalities on initial assessment in the acute setting.

Initial laboratory investigations showed a haemoglobin level of 164 g/L, with sodium and potassium within normal limits. Creatinine was 116 µmol/L, consistent with the patient’s baseline values observed during subsequent hospitalization. Liver function tests were within normal limits (AST 24 U/L, ALT 20 U/L, gamma-glutamyl transferase 26 U/L, alkaline phosphatase 66 U/L, and total bilirubin 9.8 µmol/L). Coagulation studies showed a normal prothrombin time (Quick 122%, INR 0.9) but a prolonged activated partial thromboplastin time (58 s) consistent with the known HA.

During monitoring in the emergency department, the patient developed a marked hypotensive episode. Electrocardiography showed diffuse T-wave inversions involving both limb and pre-cordial leads (*Figure 1*). High-sensitivity cardiac troponin levels were initially 35 ng/L and rose to 59 ng/L at 1 h. A repeated complete blood count demonstrated a decrease in haemoglobin of 46 g/L within 3 h. Head computed tomography excluded intracranial haemorrhage. These findings were interpreted as a type 2 non-ST-segment elevation myocardical infarction, resulting from an imbalance between myocardial oxygen supply and demand, while the hypotensive episode was attributed to acute hypovolemia, both secondary to significant bleeding.

**Figure 1:**
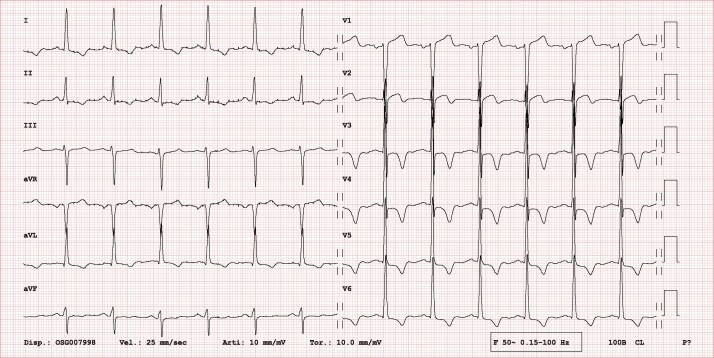
Electrocardiogram in the acute setting. The ECG demonstrates diffuse T-wave inversions involving leads I, II, aVL, aVF and V3–V6, consistent with subendocardial ischemia. Features of left ventricular hypertrophy and a left anterior fascicular block are also present.

The patient received tranexamic acid and an initial dose of FVIII plus von Willebrand Factor concentrate for the acute bleeding. This was subsequently switched to recombinant FVIII (rFVIII) concentrate once HA diagnosis (with FVIII:C 18 IU/dl) was confirmed and von Willebrand deficiency was excluded. Testing for FVIII inhibitors was negative. Repeated FVIII measurements performed later during hospitalization, outside the acute bleeding phase, showed persistently reduced levels (3 and 4 IU/dl at 72 h), consistent with a diagnosis of moderate HA. Moreover the diagnosis was confirmed by the positive family history (grandnephew affected by moderate haemophilia A, genetically characterized by the mutation F8 c.6481C>G, p.Pro2161Ala, classified as probably pathogenetic).

The patient was transferred to the intensive care unit for close monitoring. Repeated clinical examination at that stage revealed a grade 4/6 systolic ejection murmur radiating to the carotid arteries, suggestive of severe aortic stenosis. Transthoracic echocardiography demonstrated a hypertrophic left ventricle, with moderately reduced systolic function (left ventricular ejection fraction of 40% and a global longitudinal strain of −13%). The aortic valve showed severe stenosis, with a peak velocity of 5.6 m/s, a mean transvalvular gradient of 83 mmHg and an aortic valve area of 0.6 cm^2^, consistent with high-gradient severe aortic stenosis (*Figure 2*). Moderate mitral regurgitation was also present. Diastolic function showed a restrictive filling pattern (grade III) with elevated filling pressures (*E*/*E*′ septal 21, lateral 20), consistent with advanced diastolic dysfunction.

**Figure 2:**
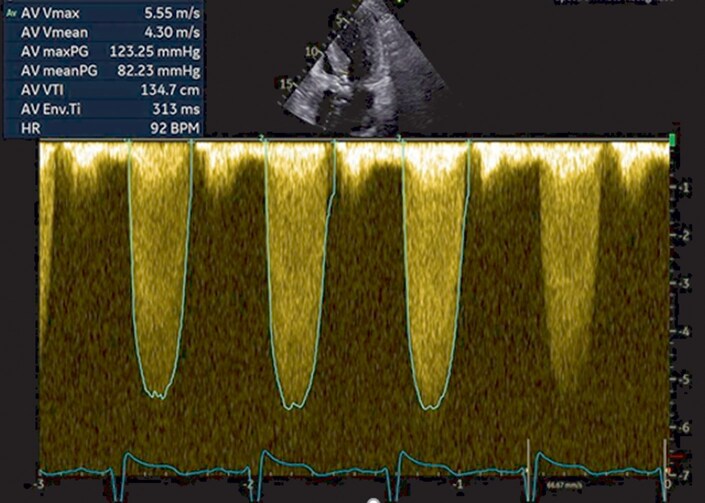
The first echocardiography. The figure shows the aortic continuous Doppler with a pattern compatible with a severe aortic valve stenosis. Below the complete report. Left ventricle: Abnormal. End-diastolic diameter 57 mm (N 40–59), indexed 31 mm/m^2^ (N 23–32). Interventricular septum 12 mm (N 6–12). End-diastolic volume 143 ml (N 60–150), indexed 78  ml/m^2^ (N 35–75). End-systolic volume 85 ml (N 20–58). Posterior wall 12 mm (N 6–11). Left ventricular mass index 158 g/m^2^ (M < 115). Relative wall thickness 0.42. Left ventricular ejection fraction 40% (biplane). Aortic valve: Abnormal. Severe stenosis with peak velocity 5.6 m/s, maximum gradient 124 mmHg, mean gradient 83 mmHg, aortic valve area 0.6 cm^2^ (indexed 0.33 cm^2^/m^2^). Moderate aortic regurgitation. Diastolic function: Grade III (restrictive). *E*/*E*′ septal 21, *E*/*E*′ lateral 20, *E*/*A* ratio 2. Global longitudinal strain (GLS) −13%. Right ventricle: Normal size and function. Basal diameter 35 mm (N ≤ 45), TAPSE 21 mm. Left atrium: Dilated. Anteroposterior diameter 48 mm (N 30–45), volume 87 ml (N 22–52), indexed 48 ml/m^2^ (N <34). Right atrium: Normal size. Area 20 cm^2^ (N ≤ 18). Interatrial septum intact. Aorta: Ascending aorta 43 mm. Mitral valve: Moderate regurgitation, no stenosis. Tricuspid valve: Mild regurgitation, no stenosis. Maximum gradient 55 mmHg. Estimated pulmonary artery systolic pressure 60 mmHg. Pulmonary valve: Mild regurgitation, no stenosis.

In this context, the initial hypotensive episode was most likely precipitated by acute hypovolemia due to blood loss, further exacerbated by the presence of severe valvular obstruction and reduced cardiac reserve, limiting the patient’s ability to maintain an adequate cardiac output. Haemodynamic status rapidly improved following intravenous fluid resuscitation, with stabilization of blood pressure and haemoglobin levels at 24 and 48 h. The patient was subsequently transferred to the internal medicine ward for further management.

A transcatheter aortic valve implantation (TAVI) procedure was discussed with the patient; however, he initially declined intervention. During hospitalization, the patient developed recurrent episodes of acute heart failure, associated with bilateral pleural effusions, which were managed with diuretic therapy (furosemide and torasemide) and, in one instance, therapeutic thoracentesis. The clinical course was further complicated by intercurrent infectious events, contributing to functional decline and a prolonged hospitalization exceeding 2 months. A severe subsequent episode of heart failure ultimately led the patient to accept the TAVI procedure.

A computer tomography angiography (CT angio) was performed and the pre-TAVI coronary angiography showed no significant coronary artery disease. In coordination with the haematology team, rFVIII was administered before the procedure at a dose of 55 IU/kg to achieve a target FVIII:C of 80–100 IU/dl. Unfractionated heparin was administered intraoperative, targeting an activated clotting time (ACT) of 200 s. A 29 mm CoreValve Evolut FX biological aortic prosthesis was successfully implanted by transfemoral access. At the end of the procedure, a loading dose of clopidogrel was administered. Notably, prior to the TAVI procedure, the patient had developed acute urinary retention with macroscopic haematuria due to traumatic catheterization. However, FVIII dosage adjustment allowed safe completion of the procedure.

Post-operatively, as suggested by the WFH, mechanical thromboprophylaxis was initiated.^10^ In the first three post-operative days, rFVIII was administered to maintain a target FVIII:C > 60 IU/dl, followed by 40–50 IU/dl from day 4 to day 7 (see *Table 1* for details). Due to the absence of significant coronary artery disease and considering the underlying bleeding risk, a multidisciplinary discussion led to the decision to limit antiplatelet therapy to a 7-day course. Heart failure therapy was progressively introduced in the post-acute phase, including the initiation and up-titration of guideline-directed medical treatment (spironolactone, ramipril, bisoprolol, torasemide, and dapagliflozin). Seven days after the procedure, the patient was transferred back to the internal medicine ward and discharged home after an additional 10 days.

**Table 1:** Pre-operative, peri-operative, and post-operative haematological approach

Day	Cardiological intervention	Haematological approach
103	Pre-operative coronary angiography + TAVI (day 0)	FVIII activity: 4 IU/dl (6h00) -> Kovaltry 55 UI/kg (3000 UI) for estimated peak activity between 100 and 130 IU/dl Coronarography by radial access + TAVI (9h00–11h00) During procedure anticoagulation with continued unfractioned heparin wit ACT target >200 s After coronary angiography, target FVIII > 60 IU/dl for the first 72 h, then 40–50 IU/dl from day +4 to day +7 FVIII activity: 72 IU/dl (11h15) FVIII activity: 52 IU/dl (16h00) -> Kovaltry 1000 UI 2× Start clopidogrel 300 mg, then 75 mg Start mechanical thromboprophylaxis
104	Day+1 post-TAVI	FVIII activity: 62 IU/dl (06h00), haemoglobin fall from 90 g/L to 83 g/L -> Kovaltry 1000 UI 3×
105	Day+2 post-TAVI	FVIII activity: 34 IU/dl (06h00), haemoglobin 85 g/L -> Kovaltry 1000 UI 4×
106	Day+3 post-TAVI	FVIII activity: 56 IU/dl (06h00), haemoglobin 85 g/L -> Kovaltry 1000 UI 4×
107	Day+4 post-TAVI	FVIII activity: 73 IU/dl (06h00), haemoglobin 82 g/L -> Kovaltry 1000 UI 3×
108	Day+5 post-TAVI	FVIII activity: 64 IU/dl (06h00), haemoglobin 82 g/L -> Kovaltry 1000 UI 3×
109	Day+6 post-TAVI	FVIII activity: 80 IU/dl (06h00), haemoglobin 86 g/L -> Kovaltry 1000 UI 3×
110	Day+7 post-TAVI Transfer to medicine ward	FVIII activity: 72 IU/dl (11h15) -> Kovaltry 1000 UI 2×/die In agreement with the cardiology team, given the high haemorrhagic risk related to the underlying social-clinical condition and the absence of significant coronary stenosis, clopidogrel was discontinued after 7 days of antiplatelet therapy (stop on day+8)
111–116	Until day +14 post-TAVI	Kovaltry 1000 UI 2×/die from day 110 (+8) until day 116 (+14)
120	Discharged from hospital	Follow-up by cardiology ambulatory

ACT, activated clotting time; FVIII, factor VIII; Kovaltry, recombinant FVIII concentrate; TAVI, transcatheter aortic valve implantation.

At the 1-year follow-up a transthoracic echocardiography demonstrated a well-functioning transcatheter aortic valve bioprosthesis, with low transvalvular gradients and only a mild paravalvular leak. Left ventricular systolic ejection fraction had improved (LVEF 59%) and no significant pulmonary hypertension was observed (*Figure 3*). These findings confirm sustained hemodynamic improvement following TAVI.

**Figure 3:**
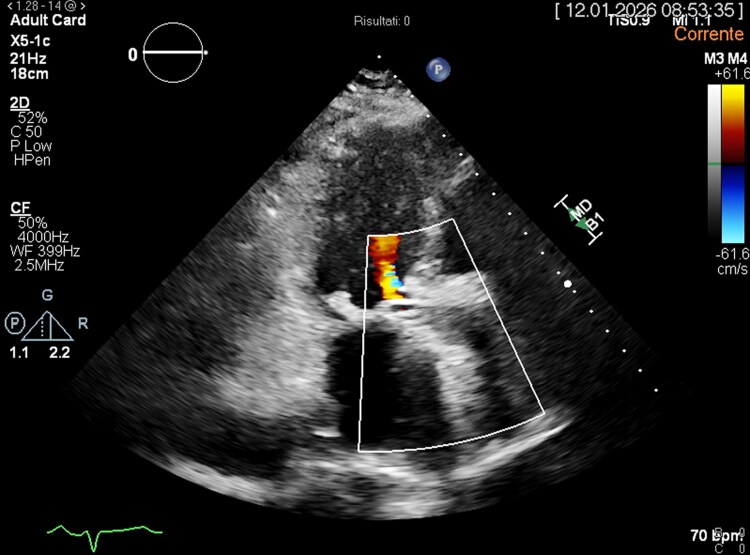
Post-TAVI echocardiography at 1 year follow-up. The figure shows a mild anterior paraprosthetic leak by colour-Doppler on an apical 3-chamber view. Below the complete report. Left ventricle: normal size (EDVi 47 ml/m^2^) with mild concentric wall hypertrophy (SIVPP 13–11 mm); regional motion and global systolic function are normal (biplane EF 59%). Left atrium: mildly dilated (LAVi 40 ml/m^2^). Aortic valve: results of transfemoral aortic revalving (TAVI) with implantation of a 29 mm Corevalve Evolut FX bioprosthesis; bioprosthesis in place, normal antegrade flowmetry (max/average G 11/5 mmHg), mild anterior paraprosthetic leak (PSAX at 2 o'clock). Mitral valve: sclerocalcic alterations of the mitral annulus extending to the base of the posterior leaflet; mild valvular stenoinsufficiency (average G 4 mmHg at heart rate 73 b.p.m.). Right heart: ventricle of normal size and kinetics (TAPSE 22 mm, *S*´ 13.7 cm/s, FAC 47%); right atrium of normal size (RAVi 22 ml/m^2^); mild tricuspid regurgitation; RV/RA gradient 25 mmHg, normal pulmonary pressures (estimated PAPs 28 mmHg). Inferior vena cava of normal size, normally collapsing. The ascending aorta is difficult to evaluate. No pericardial effusion.

## Discussion

To date, there are no well-defined guidelines for the management of patients with HA undergoing TAVI. Available literature is limited to a small number of case reports specifically describing this population. *Table 2* summarizes the main characteristics of these cases.

**Table 2:** Case reports describing the haematological approach for TAVI in patients with haemophilia a

Study	Haemophilia severity and main comorbidities	Pre-operatory management	Intra-operatory management	Post-operatory management
Merron *et al*.^12^	84 y.o. with mild haemophilia A without inhibitory history	rFVIII 2000 UI 2× week^a^	Not additional doses of rFVIII	Aspirin 100 mg 1/die
	History of quadruple CABG treated with aspirin		Heparin use is not specified	
Giuffrida *et al*.^13^	56 y.o. with severe haemophilia A without inhibitory history	rFVIII 80 UI/Kg immediately before TAVI reaching 50 UI/dl of FVIII	Bolus dose of heparin (5000 UI)	rFVIII 30 UI/Kg every 12 h reaching 30 UI/dl of FVIII level, then 25 UI/Kg for 15 days
				Heparin 100 UI/kg 1× die for 15 days
Rodríguez López *et al*.^14^	54 y.o with mild haemophilia A without inhibitory history	rVIII 50 UI/Kg 30 min before TAVI	Bolus dose of heparin (5000 UI)	rFVIII substitution: 3 h post-TAVI: rFVIII 10 UI/kg with measured FVIII of 110 UI/dl 8 h after the first administration: rFVIII every 8 h targeting FVIII activity of 80 UI/dl for 48 h after first administration 48 h after first administration: rFVIII every 12 h targeting FVIII activity of 50 UI/dl up to 7 days 7 days after first administration: rFVIII every 12 h targeting 30 UI/dl and desmopressin on alternate days until 12 days of total treatment Start heparin 40 mg/die 1 day post-op until discharge (14 days) Start aspirin 100 mg/die 1 day post-op

CABG, coronary artery bypass graft; FVIII, factor VIII; rFVIII, recombinant factor VIII; TAVI, transcatheter aortic valve implantation.

^a^Following a CABG, antiplatelet therapy with aspirin was indicated, which subsequently led to gluteal bleeding. Therefore, prophylaxis with FVIII was introduced, targeting activity levels >30 UI/dl, with no further bleeding episodes.

In the case report by Merron *et al.*, an 84-year-old patient with mild HA and a history of four coronary artery bypass grafts is described. The patient was already on aspirin and required prophylaxis with rFVIII to tolerate antiplatelet treatment due to a history of gluteal bleeding. For the TAVI procedure, no adjustment of the patient’s usual FVIII regimen (FVIII 2000 IU twice daily) was made and it remains unclear whether pre-operative heparin was administered.^12^ Giuffrida *et al.*, described a case of a 56-year-old patient with severe HA who did not require regular replacement therapy. A pre-operative bolus of rFVIII was administered to achieve a target FVIII activity >50 IU/dl, followed by 5000 IU of intravenous heparin during the procedure. For the following 15 days, rFVIII replacement and low-molecular-weight heparin (LMWH) were prescribed until discharge.^13^ In the case report by Rodriguez *et al.*, the patient had mild HA and received peri-operative heparin along with a strict post-operative FVIII replacement protocol, including the use of desmopressin (likely to prevent the development of inhibitory antibodies against FVIII).^14^ In all three reported cases, no haemorrhagic complications were observed throughout the peri-operative course.

In our patient, replacement therapy with rFVIII was initiated with a target FVIII activity between 80–100 IU/dl. During the procedure, unfractionated heparin was administered to achieve an ACT target of 200 s. Post-operatively, FVIII replacement therapy was maintained above 60 IU/dl for the first 72 h, followed by 40–50 IU/dl from day 4 to day 7, consistent with the strategy proposed in the WFH guidelines. Subsequently, it was decided to continue low-dose FVIII replacement for an additional 7 days as suggested in guidelines (see *Table 2* for details). Simultaneously, mechanical thromboprophylaxis with compression stockings was initiated, and antiplatelet monotherapy with clopidogrel was limited to one week following a multidisciplinary discussion, considering the absence of significant coronary artery stenosis and the high risk of falls in a patient with chronic alcohol abuse.

No procedure-related bleeding complications were observed during the post-operative course. Episodes of macrohaematuria related to bladder catheter placement did not interfere with the TAVI procedure. Although antiplatelet therapy was discontinued, the patient remained asymptomatic at the one-year follow-up, with no clinical evidence of thromboembolic events.

## Conclusions

Although experience is limited to case reports, published evidence indicates that haemophilic patients should not be excluded from invasive procedures if well managed through an interdisciplinary approach. In particular, TAVI can be a safe procedure by mitigating the bleeding risk with FVIII replacement during the peri-operative period. Furthermore, an interdisciplinary team evaluation (including haematologists, anaesthetists and cardiologists) is essential to develop a tailored antithrombotic strategy for the specific clinical situation. In this perspective, our case emphasizes that no significant bleeding complications were observed during the procedure itself, while the only bleeding episode was related to a low-risk procedure, such as bladder catheter placement.

## Data Availability

All data relevant to the study are included in this article.

## Lead author biography

Vincent Roger Gianolli earned his medical degree from the University of Lausanne in 2020. Following graduation, he began his post-graduate training in Payerne and Bellinzona (Switzerland), where he obtained foundational experience in internal medicine. He is currently serving as a medical resident at the Lausanne University Hospital.

## Notes

**Consent:** Informed consent by the patient has been obtained for this case.
